# Neutrophil death pathways in myocardial infarction: the balance between injury and repair

**DOI:** 10.3389/fimmu.2026.1875622

**Published:** 2026-07-01

**Authors:** Wenxi Yu, Chen Chen, Ailin Hou, Lintong Yu, Zhiyan Ma, Ningning Wang, Xiaojuan Ma, Dazhuo Shi

**Affiliations:** 1 Beijing University of Chinese Medicine, Beijing, China; 2 Xiyuan Hospital, China Academy of Chinese Medical Sciences, Beijing, China; 3 Macau University of Science and Technology, Macao, Macao SAR, China

**Keywords:** myocardial infarction repair, NETosis (neutrophil extracellular traps), platelet-neutrophil crosstalk, programmed neutrophil death, thromboinflammation

## Abstract

Following acute myocardial infarction (AMI), neutrophils rush to the damaged heart tissue. Their presence is critical, and how they die influences whether the heart heals or suffers further injury. This outcome depends on specific cell death pathways of neutrophils, including NETosis, apoptosis, and autophagy. NETosis can be harmful when neutrophils release sticky, web-like structures (NETs) filled with toxic enzymes, particularly during early thromboinflammatory amplification. These webs trap platelets and trigger clotting, which blocks blood vessels and worsens heart damage. In contrast, timely neutrophil apoptosis is a quiet, controlled death that signals cleanup cells (macrophages) to remove debris and start tissue repair, although apoptotic signaling in other cardiac cell types or inappropriate time windows may be detrimental. Autophagy acts as a regulator, helping determine which of these paths the cell takes. Furthermore, platelets could modulate these specific cell death pathways by releasing soluble mediators (e.g., P-selectin, HMGB1, polyP, CXCL4), promoting NETosis while suppressing apoptosis to exacerbate ischemic myocardial injury. Some anti-inflammatory strategies could fail if broad immune suppression inadvertently disrupts reparative neutrophil apoptosis and efferocytosis. Future therapies could aim to precisely block pathological NETosis or support timely neutrophil apoptosis to limit injury and improve heart recovery.

## 1 Introduction

Acute myocardial infarction (AMI) triggers a massive immune response. Neutrophils are the first immune cells to arrive at the injured heart, recruited by distress signals released from dying myocardial cells. Their primary job is to clear away dead tissue so healing can begin. However, if these cells remain active for too long, they release toxic chemicals that exacerbate myocardial injury and drive adverse cardiac remodeling.

Recent research shows that neutrophils are not just simple soldiers; the specific way they die influences the heart’s recovery (1). This concept is known as programmed cell death (PCD). We now understand that different death pathways, such as NETosis, apoptosis, and autophagy, have distinct effects on the heart.

1. NETosis (Context-dependent thromboinflammatory driver): In this process, neutrophils expel their DNA to form Neutrophil Extracellular Traps (NETs). While meant to trap bacteria, in a sterile heart attack, these traps can drive inflammation and clotting (thromboinflammation), blocking blood vessels and expanding the area of injury, especially during the early inflammatory phase (2, 3).
2. Apoptosis (Resolution-associated when timely cleared): This is a programmed “suicide” where the cell packages its contents safely. This signals macrophages to eat the dying neutrophil (a process called efferocytosis), which then triggers the release of anti-inflammatory signals to resolve inflammation and build a stable scar (4).
3. Autophagy (Regulatory): Emerging evidence implicates autophagy in modulating neutrophil death modalities (e.g., NETosis vs. apoptosis), thereby impacting post-MI recovery.

The importance of these pathways is supported by genetic data. A recent study proposed a 9-gene neutrophil-derived programmed cell death signature (NPCDS), including MDM2, PTK2B, MYH9, IVNS1ABP, MAPK14, GNS, MYD88, TLR2, and CFLAR, that may support AMI prediction and stratification into NPCDS-based molecular subtypes with distinct neutrophil inflammatory profiles (5).

Treating this inflammation has proven difficult. A major “clinical paradox” exists: broadly suppressing the immune system often fails to fix the heart. For instance, colchicine has shown benefit in selected post-MI or chronic coronary disease populations in COLCOT and LoDoCo2, whereas the more recent CLEAR SYNERGY/OASIS-9 trial did not significantly reduce cardiovascular events after acute MI (6–8). The IL-1 pathway further illustrates that different modes of IL-1 blockade may have distinct biological and clinical consequences (9). Anakinra, a recombinant IL-1 receptor antagonist that blocks IL-1α and IL-1β signaling through IL-1R1, reduced inflammatory biomarkers in non-ST-elevation acute coronary syndromes but did not establish a clear clinical benefit (10). In contrast, canakinumab, a monoclonal antibody selectively targeting IL-1β, reduced recurrent cardiovascular events at the 150-mg dose in CANTOS, although this benefit was accompanied by an increased risk of fatal infection (11). Thus, simply suppressing inflammatory signaling is unlikely to be sufficient unless timing, target specificity, dose, safety, and preservation of reparative immune programs are considered.

**Figure 1:**
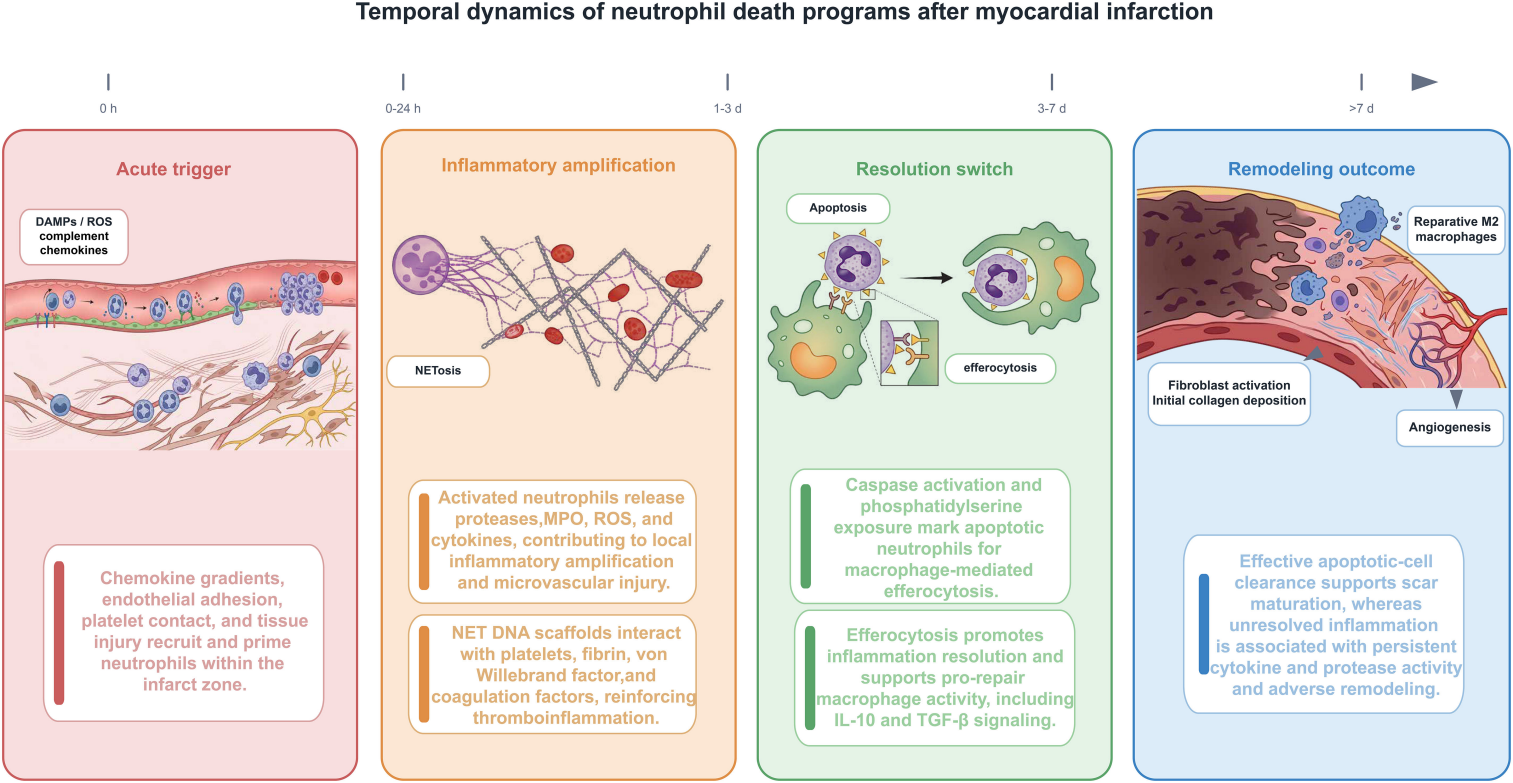
Temporal dynamics of neutrophil death pathways after myocardial infarction. The schematic summarizes stage-specific shifts in neutrophil recruitment, NETosis/protease release, apoptosis/efferocytosis, and late remodeling after MI. MI, myocardial infarction; NETosis, neutrophil extracellular trap formation; DAMPs, damage-associated molecular patterns; ROS, reactive oxygen species; MPO, myeloperoxidase.

This review examines how the balance between neutrophil NETosis, apoptosis, and autophagy changes over time and shapes the outcome of a heart attack (**Figure 1**). We propose that precision therapies should selectively restrain destructive NETosis while preserving or enhancing timely neutrophil apoptosis and efferocytosis (**Table 1**).

**Table 1:** Modulation of neutrophil death pathways influences post-myocardial infarction prognosis: mechanisms and therapeutic implications.

Authors/refs	Targeted cells	Study subjects	Results	Mechanisms
Mingjun Du., et al. (18)	Neutrophils	Male C57BL/6 mice	Ventricle histological structure preservation ↑ Myocardium integrity ↑ Infarct size ↓ Myocardial enzyme levels in serum ↓ Inflammatory cytokine secretion ↓ Cardiomyocyte apoptosis ↓ Overall cardiac function improvement ↑	Inhibition of PAD4 reduces NETosis
Chunxia Chen., et al. (22)	Neutrophils	Male C57BL/6 mice Wistar rats	Gut microbiota migration to blood ↑ (post-I/R) Cardiac I/R injury severity ↑ (aggravated by gut microbiota) Myocardial cell/myocardial microvascular endothelial cell apoptosis ↑ (directly induced by NETs)	Gut microbiota migrate to the bloodstream post-I/R, stimulate NETs formation
Kristine Y DeLeon-Pennell., et al. (33)	Neutrophils, Macrophages	Male and female C57BL/6J wild-type and MMP-9-null mice (*in vivo*); macrophages and neutrophils isolated from C57BL/6 mice (*in vitro*)	Macrophage phagocytic capacity ↓ (with MMP-9 or CD36-blocking peptide) Apoptotic neutrophil accumulation in MMP-9 null group ↑ (vs. wild-type) Neutrophil apoptosis ↓ (via MMP-9 stimulation)	MMP-9 proteolytically degrades CD36, impairing macrophage phagocytosis/efferocytosis and delaying apoptotic neutrophil clearance during cardiac remodeling.
Rugmani Padmanabhan Iyer., et al. (25)	Neutrophils	Male C57BL/6J mice (*in vivo*) neutrophils isolated from C57BL/6 mice (*in vitro*)	Left ventricular (LV) dilation ↑ (MMP-12 inhibition group vs. control) LV function ↓ Pro-inflammatory cytokines (IL1r1, IL6ra, IL11, Cxcr5) ↑ (prolonged upregulation)	MMP-12 inhibition impairs CD44-HA interactions, leading to suppressed neutrophil apoptosis; CD44 transcription/translation effects are most evident at day 7 post-MI.
Mikhail A Kolpakov., et al. (36)	Neutrophils	Female and Male C57BL/6J mice	Day 2: reduced cardiomyocyte apoptosis and early functional improvement, but infarct area was similar between Par4−/− and WT mice. Chronic MI (days 7-28, Par4−/− vs. WT): cardiac rupture incidence ↑; mortality ↑; infarct expansion ↑; cardiac hemorrhage ↑; inflammation resolution ↓; neutrophil apoptosis ↓. Adoptive transfer experiments support a neutrophil-intrinsic PAR4 role.	PAR4 promotes neutrophil apoptosis during post-MI healing; LXA4 dynamics are delayed rather than uniformly suppressed.
Fabrizio Montecucco., et al (44)	Neutrophils	Male C57Bl/6 mice(*in vivo*) Langendorff heart model(ex vivo) Human peripheral blood mononuclear cells and T cell leukemia cell line Jurkat	Myocardial infarct size ↓ (Nampt inhibitor FK866-treated mice) Neutrophil infiltration in infarcted hearts ↓ ROS generation in hearts ↓ Circulating CXCL2 levels ↓ (serum from FK866-treated mice) Neutrophil migration capacity ↓ (primed by FK866-treated sera) CXCL8 release by human PBMCs and Jurkat cells ↓ (via FK866, SIRT inhibitors, or SIRT6 silencing) No protective effect in Langendorff ex vivo model (no leukocytes) →	Nampt inhibition suppresses SIRT6 activity, thereby reducing CXCL2/CXCL8 production and attenuating neutrophil chemotaxis
Lili Bao., et al (55)	Neutrophils, Macrophages	10-week-old female Sprague Dawley rats (*in vivo*); bone marrow-derived neutrophils/macrophages and rat myocardial cell line H9C2 (*in vitro*)	Cardiac function ↑ (eNABHAL-treated MI rats); inflammation in infarcted region ↓; macrophage efferocytosis/apoptotic cell clearance ↑	eNABs target infarct-associated macrophages, enhancing efferocytosis and inflammation resolution, promoting tissue repair
Ricardo A García., et al. (56)	Neutrophils, Macrophages	male C57BL/6 mice and male Sprague Dawley rats(*in vivo*) Langendorff-perfused ex vivo system(rats hearts)	Mouse survival rate ↑ LV scar area ↓ LV wall thickness preservation ↑ Macrophage proresolution markers ↑ (arginase-1 mRNA, CD206) Neutrophil apoptosis ↑ IL-10 and MCP-1 gene expression ↑ Viable myocardium in rats ↑ LV ejection fraction ↑ LV remodeling attenuation ↑	FPR2 agonism (BMS-986235): Drives macrophages toward a proresolution phenotype. Enhances macrophage phagocytosis and neutrophil apoptosis.
Michael Horckmans., et al. (46)	Neutrophils, Macrophages	Female 10- to 12-week-old C57BL/6J mice (*in vivo*) Murine bone marrow-derived macrophages and human monocytes(*In vitro*)	Cardiac function ↓ (neutrophil-depleted mice) Myocardial fibrosis ↑ Heart failure progression ↑ M2c macrophage marker MerTK (phagocytosis receptor) ↓ Apoptotic cell clearance ↓ (TUNEL-positive cells ↑) Macrophage reparative phenotype restored by neutrophil secretome or NGAL →	Neutrophils secrete NGAL to: Polarize macrophages toward M2c reparative phenotype (↑ MerTK). Enhance macrophage efferocytosis (clearance of apoptotic cells).
Ganesh V Halade., et al. (57)	Neutrophils, Macrophages	Male C57BL/6J mice	Acute heart failure signs ↑ (CAP + MI vs. MI-control); CD47 expression ↑; pre-MI splenic neutrophil activation ↑; post-MI reparative neutrophils/macrophages ↓; activated neutrophils (Ly6G+) in LV post-MI ↑; macrophage phagocytosis ↓; splenocardiac leukocyte apoptosis ↑; prostaglandins/thromboxanes ↑; cardioprotective EETs ↓; pro-inflammatory cytokines ↑/reparative cytokines ↓	CAP disrupts resolution process: Upregulates CD47 → impairs macrophage efferocytosis. Pre-activates splenic neutrophils → amplifies inflammatory infiltration. Reduces reparative macrophage/neutrophil subsets → impairs tissue repair. Delays leukocyte apoptosis clearance → unresolved inflammation.
Akrivi Chrysanthopoulou., et al. (21)	Neutrophils/Platelets	Human STEMI thrombi and ex vivo neutrophil-platelet models; male C57BL/6J mice (FeCl3 arterial thrombosis model)	IFN-λ1/IL-29 reduced NET release and cytoplasmic TF in human STEMI-related neutrophil models. Platelet-derived polyP was present in STEMI thrombi and triggered NET formation through mTOR inhibition and autophagy induction; IL-29 counteracted polyP-induced mTOR inhibition/autophagy and suppressed NET formation. IFN-λ2/IL-28A reduced thrombus formation *in vivo*.	Platelet-derived polyP promotes autophagy-dependent NET formation, whereas IFN-λ signaling restrains NETosis-driven thromboinflammation.
Julia Etulain., et al. (63)	Neutrophils, Platelets	Neutrophils from P-selectin(ΔCT/ΔCT) mice and P-selectin(-/-) mice Human neutrophils incubated with platelets	Platelet-induced NETosis inhibited by anti-P-selectin aptamer/anti-PSGL-1 antibody P-selectin^-^/^-^ platelets failed to induce NETosis Soluble P-selectin directly promoted NETosis P-selectin(ΔCT/ΔCT) neutrophils showed priming (↑ basal citrullination) and enhanced NETosis upon stimulation.	P-selectin (cellular/soluble) binds PSGL-1 on neutrophils, promotes NET formation and drives thrombo inflammation
N Maugeri., et al. (60)	Neutrophils, Platelets	Human subjects: thrombi from AMI patients; neutrophils and platelets from healthy donors wild-type Myd88-/- and Rage-/- mice: neutrophils from the bone marrow	NET formation in coronary thrombi ↑ (associated with activated platelets) Platelet-induced NET generation ↓ (with HMGB1 antagonists or Hmgb1-/- platelets) Mitochondrial potential depletion prevented ↑ (via HMGB1 exposure) Autophagosome formation ↑ Neutrophil survival ↑ (HMGB1-mediated) Autophagic flux blockade → NET formation ↓	Activated platelets release HMGB1 → binds to neutrophil RAGE. Triggers autophagy Sustained autophagy prolongs neutrophil survival and drives NET extrusion.
Huijuan Dou., et al. (65)	Neutrophils, Platelets	Lnk-/- mice Jak2VFLnk+/-mice Human LNK(TT) vs. LNK(CC) iPSC-derived neutrophils/platelets	Lnk Deficiency (Mice): ↑ NETosis, accelerated thrombosis & atherosclerosis PAD4 KO reversed thrombosis/NETosis E06-scFv (anti-OxPL) reversed NETosis/thrombosis Human LNK(TT): ↑ NETosis with LNK(TT) platelets vs. LNK(CC)	Increased platelet OxPL exposure/release leads to NETosis and accelerated thrombosis in hematopoietic LNK deficiency
H Hartwig., et al. (67)	Neutrophils, Platelets	Platelet-deficient mice *In vitro* experiments: Neutrophils incubated with platelets	Neutrophil apoptosis ↑ (in platelet-deficient mice or with PF4 immunodepletion) Annexin V binding (apoptosis marker) on circulating neutrophils ↑ (with anti-PF4 antibodies in femoral artery ligation model) NET formation ↑ (PF4-mediated proatherogenic effect) No anti-apoptotic effect observed with CCL5, serotonin, or TGFβ →	CXCL4 (PF4) released from platelet α-granules upon activation Functional effects: Suppresses neutrophil apoptosis (dose-dependent). Promotes NET formation, contributing to thromboinflammatory pathology).
Tanja Vajen., et al. (68)	Neutrophils, Monocytes/Macrophages	male C57BL/6 mice	Infarct size ↓ (MKEY vs. sMKEY) Heart function preservation ↑ (e.g., ejection fraction, ventricular pressure) Inflammatory cell infiltration ↓ (neutrophils, monocytes/macrophages in infarct zone)	MKEY(engineered inhibitory peptide) disrupts CCL5-CXCL4 heterodimer formation, which: Attenuates leukocyte recruitment (neutrophils and monocytes/macrophages) to the infarcted heart. Inhibits NETosis

AMI, Acute myocardial infarction; CAP, Carprofen; CXCL, Chemokine ligand (e.g., CXCL2, CXCL4, CXCL8); eNABs, Engineered neutrophil apoptotic bodies; EETs, Epoxyeicosatrienoic acids; FPR2, Formyl peptide receptor 2; HMGB1, High mobility group box 1; I/R, Ischemia/reperfusion; LV, Left ventricular; MCP-1, Monocyte chemoattractant protein-1; MMP, Matrix metalloproteinase (e.g., MMP-9, MMP-12); NETs, Neutrophil extracellular traps; NPCDS, Neutrophil-derived programmed cell death signature; PCD, Programmed cell death; NGAL, Neutrophil gelatinase-associated lipocalin; PAR4, Protease-activated receptor 4; polyP, Platelet-derived inorganic polyphosphate; RAGE, Receptor for advanced glycation end products; ROS, Reactive oxygen species; SIRT6, Sirtuin 6; STEMI, ST-segment elevation myocardial infarction; TF, Tissue factor.

To clarify the scope of this review, other regulated cell-death programs should also be acknowledged. Pyroptosis, necroptosis, and ferroptosis are increasingly recognized in heart disease (12), and experimental MI studies support roles for RIP3-dependent necroptosis, and GPX4-associated ferroptosis in adverse remodeling or cardiomyocyte injury (13, 14). However, current evidence for these pathways after MI is concentrated largely in cardiomyocytes, endothelial cells, macrophages, and other cell populations rather than neutrophil-specific post-MI biology. Gasdermin D-dependent NET formation suggests potential crosstalk between pyroptotic machinery and NETosis, but direct evidence in MI-related neutrophil death remains limited (15). Therefore, this review focuses on NETosis, apoptosis, and autophagy while acknowledging that their interactions with other death programs require further study.

## 2 NETosis: a thromboinflammatory driver in myocardial injury

NETosis generates Neutrophil Extracellular Traps (NETs), which are weblike structures composed of nuclear DNA, histones (e.g., citrullinated histone H3Cit), and granular proteins (e.g., myeloperoxidase, neutrophil elastase). While NETs serve a protective role in infection by trapping pathogens, they act as key drivers of thromboinflammation in thrombotic disorders through two interconnected mechanisms: (1) Prothrombotic Scaffolding: The DNA backbone of NETs binds von Willebrand factor (vWF) and fibrinogen, forming a structural scaffold for thrombi. Histones (particularly H3/H4) anchor platelets to injury sites through interactions with vWF and activate platelets via Toll-like receptors (TLR2/4), enhancing platelet aggregation. Simultaneously, histones inhibit thrombomodulin-dependent protein C activation, thereby promoting thrombin generation. Furthermore, NET-associated elastase amplifies coagulation by inactivating tissue factor pathway inhibitor (TFPI). (2) Inflammatory Amplification: NET components (e.g., histones, DNA) act as damage-associated molecular patterns (DAMPs) that activate immune cells via TLRs, triggering the immediate release of pro-inflammatory mediators such as TNF-α and CXCL7. Concurrently, some components (e.g., histones, elastase) directly damage endothelium, inducing endothelial secretion of vWF and P-selectin. This recruits additional platelets and neutrophils, creating a self-sustaining inflammatory cascade (2).

Clinical evidence confirms that NETs are widely present in human thrombus specimens (16) and their serum levels correlate positively with acute coronary syndrome severity (17). Thus, distinct mechanisms regulating NETosis in MI offer promising therapeutic targets against thromboinflammation. For example: the enzymatic control of chromatin decondensation via PAD4, immunomodulatory interferon signaling pathways (IFN-λ), and gut microbiota-mediated neutrophil activation. These intersecting axes not only drive infarct pathogenesis but also offer actionable targets for precision immunotherapies.

### 2.1 PAD4-driven NETosis

The calcium-dependent enzyme peptidyl arginine deiminase-4 (PAD4) mediates histone H3 citrullination (CitH3), an essential post-translational modification required for NET formation. Cardiac PAD4 expression escalates post-MI, correlating with neutrophil infiltration. In a male C57BL/6 mouse MI model, pharmacological PAD4 inhibition using GSK484 demonstrates cardioprotection by reducing infarct size, lowering concentrations of myocardial enzymes(CK-MB, LDH, and cTnT), and suppressing CitH3 expression. Notably, PAD4 ablation disrupts NET formation-evidenced by diminished extracellular DNA scaffolds colocalized with diffuse neutrophil elastase (NE)- without impairing physiological neutrophil recruitment, suggesting selective NETosis inhibition mitigates inflammatory injury (18).

### 2.2 IFN-λ-mediated NET suppression

Type III interferons (IFN-λ1/IL-29 in humans; IFN-λ2/IL-28A in mice) not only exert well-known antiviral effects but also exhibit novel thromboinflammatory regulation effects. Because mouse Ifnl1 is a pseudogene and IFN-λ1/IL-29 is not expressed in mice, murine studies commonly use IFN-λ2/IL-28A as the functional counterpart (19). In ex vivo human STEMI models, IFN-λ1/IL-29 treatment reduces cytoplasmic tissue factor (TF)- the primary initiator of blood coagulation and a bioactive component of NETs- while inhibiting NET release. IFN-λ2/IL-28A has been shown to exert strong antithrombotic potential *in vivo*, a finding consistent with its established role in attenuating neutrophil proinflammatory activation and migration in various disease models (20). Collectively, this regulatory axis positions IFN-λ signaling as an endogenous checkpoint against pathological NETosis-driven thromboinflammation (21).

### 2.3 Gut microbiota-dependent NETosis and microbial translocation

Emerging evidence links gut microbiota-dependent immune activation to exacerbated NETosis in cardiac ischemia/reperfusion (I/R) injury. Comparative analysis reveals: Gut microbiota-depleted mice exhibit reduced neutrophil infiltration and CitH3+ NET levels compared with the DNase I-treated positive control group, where DNase I was administered to enzymatically degrade NET scaffolds. The microbiota-depleted models exhibited reduced cardiomyocyte apoptosis, as evidenced by decreased TUNEL-positive cells, suggesting attenuated cardiac damage. *In vitro* validation demonstrated that NET-enriched supernatants induce pro-apoptotic BAK upregulation and sustain caspase-3 activation in both neonatal rat ventricular myocytes (NRVMs) and cardiac microvascular endothelial cells (CMECs) (22). These findings support a gut microbiota/NET axis, while the responsible microbial products and translocation mechanisms require further clarification.

## 3 Apoptosis: an orchestrator of inflammation resolution

Neutrophils—short-lived innate immune cells—undergo spontaneous apoptosis to limit inflammatory cascades, while their efferocytosis-mediated clearance coordinates inflammatory resolution and tissue repair post-MI. By maintaining plasma membrane integrity—a hallmark distinguishing it from necrotic death—apoptotic neutrophils prevent cytotoxic granule release (e.g., myeloperoxidase), thereby preventing collateral tissue damage (3). Concurrently, they secrete annexin A1 and lactoferrin to suppress further neutrophil recruitment while attracting monocytes and macrophages for debris clearance (4). Macrophage-mediated efferocytosis of apoptotic neutrophils clears apoptotic cells and reprograms macrophages toward an anti-inflammatory phenotype via TGF-β/IL-10 secretion (4, 23).

In standard murine/canine MI models, neutrophil infiltration is robust but transient, declines after 3 days, and is very low by 7 days after reperfusion, temporally aligning with inflammatory resolution (24). However, the ischemic microenvironment can subvert this clearance program by prolonging neutrophil lifespan, a dysregulation clinically correlated with acute coronary syndrome (ACS) severity (3). Flow cytometric analysis of ACS patients reveals delayed neutrophil apoptosis compared to stable angina pectoris (SAP) or healthy controls. Serum transfer experiments demonstrated that ACS patient serum-induced marked apoptosis resistance in healthy donor PMNs versus SAP/control serum. Subsequent *in vitro* validation identified proinflammatory cytokines IFN-γ, GM-CSF, and IL-1β as potent inhibitors of neutrophil apoptosis (3).

Critically, caspase-3 activation should be interpreted according to cell type and timing. During the acute phase, apoptotic signaling in cardiomyocytes can contribute to infarct injury, whereas in infiltrating neutrophils caspase activation is part of apoptosis and subsequent efferocytic clearance (25, 26). This temporal and cellular specificity creates a therapeutic paradox: nonselective suppression of apoptotic signaling may attenuate early cardiomyocyte death but may also risk impairing neutrophil clearance and stable scar formation. Thus, pharmacological strategies should avoid global apoptosis blockade and instead selectively accelerate timely neutrophil apoptosis and efferocytosis, thereby mitigating persistent inflammation while preserving endogenous repair mechanisms essential for post-MI recovery.

### 3.1 MMP-9 and MMP-12: dual regulators of neutrophil apoptosis and cardiac repair post-MI

Matrix metalloproteinase (MMP)-9 is a key enzyme modulating post-MI left ventricular remodeling; classical MMP-9 deletion studies linked it to LV enlargement, collagen accumulation, and angiogenesis after experimental MI (27, 28). Clinical studies further show that circulating MMP-9 changes after acute MI are associated with LV remodeling and post-MI prognosis (29, 30). Reviews of post-MI matrix remodeling further support MMP-9 as a context-dependent mediator of extracellular matrix turnover, leukocyte trafficking, and ventricular dilation (31, 32). More recently, MMP-9 has been shown to critically regulate neutrophil apoptosis and macrophage efferocytosis during cardiac repair. *In vitro*, recombinant MMP-9 suppressed neutrophil apoptosis, as indicated by reduced caspase-9 expression (33). In MMP-9-/- mice, increased apoptotic neutrophils were observed at day 7 post-MI, evidenced by higher caspase-3 activation and TUNEL-positive neutrophils compared to the wild-type (WT) group (33). Proteomic profiling of infarcted myocardium identified CD36, a macrophage phagocytosis receptor, as a candidate MMP-9 substrate. MMP-9 deficiency resulted in sustained CD36 expression, correlating with enhanced macrophage phagocytic capacity. Mechanistically, MMP-9-mediated CD36 degradation decreases macrophage-mediated phagocytosis of apoptotic neutrophils, as shown by similar reductions in phagocytic capacity after MMP-9 stimulation, CD36 blockade, or combined MMP-9/CD36 inhibition. In parallel, MMP-9 directly regulates neutrophil apoptosis *in vitro*: it decreased caspase-9 expression while increasing caspase-3 expression, with the latter interpreted by the original authors as a possible compensatory response to reduced caspase-9. Because CD36 inhibition increased caspase-3 and Xiap expression without affecting caspase-9, the authors interpreted the direct apoptotic effect of MMP-9 as independent of CD36 degradation (33). Given that excessive MMP-9 activity can promote ECM degradation and adverse ventricular remodeling (27, 28, 31, 32), while also impairing macrophage efferocytosis of apoptotic neutrophils (33), therapeutic interventions should restrain excessive MMP-9 activity.

**Figure 2:**
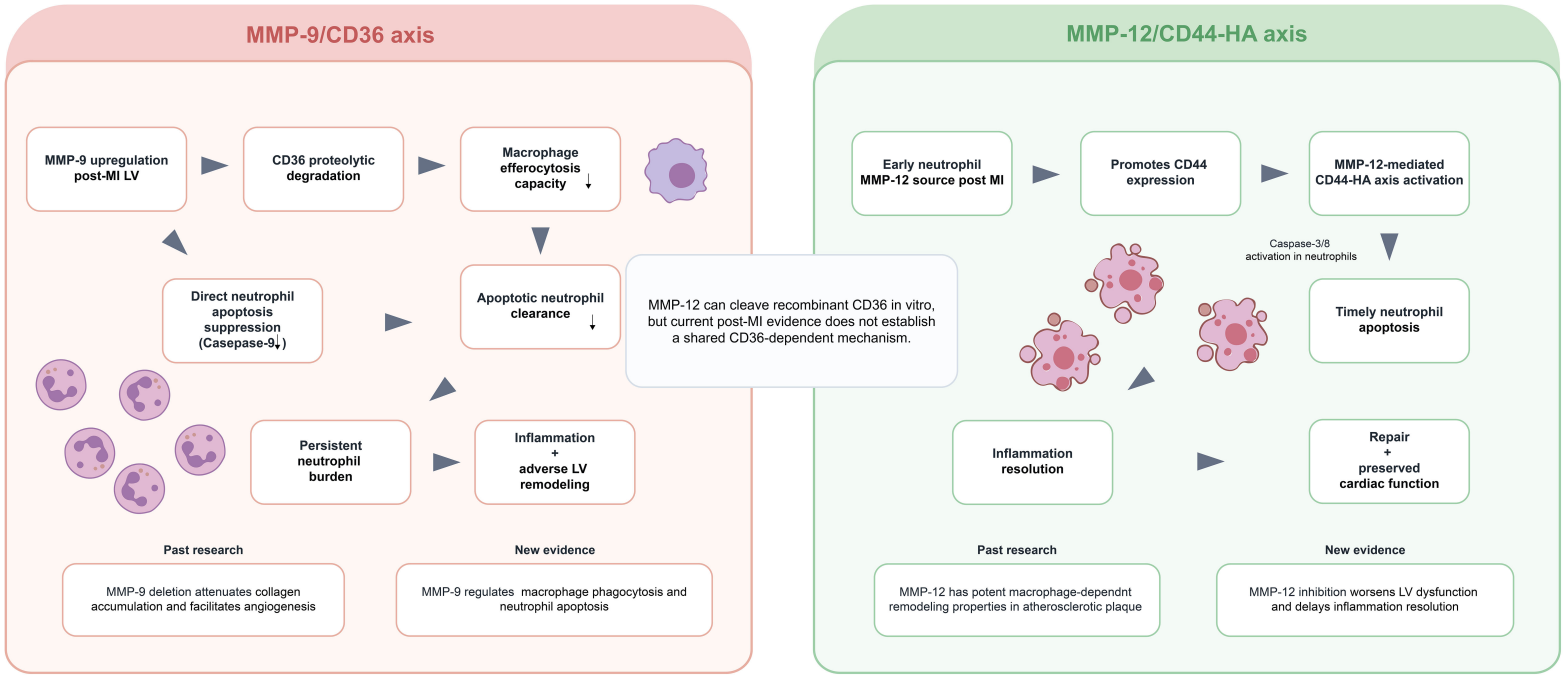
Context-dependent roles of MMP-9 and MMP-12 in post-MI neutrophil apoptosis and repair. MMP-9 promotes CD36 degradation, reduces macrophage phagocytosis/efferocytosis, directly suppresses neutrophil apoptosis *in vitro*, and may prolong inflammation, whereas MMP-12 is linked to CD44-hyaluronan signaling, neutrophil apoptosis, and inflammation resolution. MI, myocardial infarction; MMP, matrix metalloproteinase; CD36, cluster of differentiation 36; HA, hyaluronan; CD44, cluster of differentiation 44; ECM, extracellular matrix.

MMP-12, also known as macrophage metalloelastase, is an elastin-degrading MMP originally characterized in macrophages and later implicated in macrophage-rich inflammatory matrix remodeling and vascular lesion biology (34, 35). Although the MMP-9 study found that MMP-12 can cleave recombinant CD36 *in vitro*, this observation does not establish that MMP-12 regulates post-MI cardiac repair through the same CD36-dependent pathway. The available MMP-12 study instead supports a CD44-hyaluronan (HA) axis through which MMP-12 deficiency worsens cardiac outcomes by impairing neutrophil apoptosis and amplifying inflammatory responses (25). The study identified neutrophils as an unexpected early source of MMP-12, traditionally considered macrophage-derived, in infarcted myocardium, evidenced by its pronounced expression in day 1 post-MI neutrophils compared to baseline controls. Functional studies demonstrate MMP-12’s cardioprotective role: pharmacological inhibition of MMP-12 (MMP-12i) exacerbates left ventricular dysfunction (end-diastolic volume, ejection fraction) and adverse remodeling (hypertrophy index, ECM degradation) compared to saline controls, concomitant with impaired neutrophil apoptosis. Mechanistically, MMP-12i disrupts CD44-hyaluronan (HA) interactions, suppressing CD44 transcription and translation most clearly at day 7 post-MI, which coincides with pathological HA accumulation and sustained pro-inflammatory signaling (IL1R1, IL6RA, IL11, CXCR5 upregulation). This CD44-HA axis impairment directly correlates with attenuated neutrophil apoptosis, validated *in vitro* by active MMP-12 stimulation of CD44 mRNA and caspase-3/8 activation in wild-type neutrophils. These context-dependent MMP-9 and MMP-12 mechanisms are summarized in **Figure 2**.

### 3.2 Neutrophil-intrinsic PAR4 orchestrates apoptosis-driven inflammation resolution in post-MI healing

Beyond its canonical role in platelet activation, protease-activated receptor 4 (Par4)—a thrombin-sensitive G protein-coupled receptor (GPCR)—emerges as a critical regulator of neutrophil apoptosis and inflammatory resolution during post-MI cardiac repair. Par4 mRNA/protein expression escalates in infarcted myocardium and cytokine-stimulated cardiomyocytes. Genetic ablation of Par4 reveals temporally divergent effects: during acute MI (day 2), Par4^-^/^-^ mice exhibit reduced cardiomyocyte apoptosis and some early functional improvement, whereas infarct size, measured as the percentage of LV circumference, was similar between WT and Par4^-^/^-^ mice at day 2 post-MI (36). However, this early benefit reverses in chronic phases (days 7-28), with Par4^-^/^-^ mice developing exacerbated cardiac dysfunction, infarct expansion, increased myocardial rupture rates, and higher mortality. Histopathological analysis demonstrates impaired healing marked by increased necrotic areas, hemosiderin deposition, reduced collagen density, and diminished SMA^+^(α-actin) myofibroblast infiltration—key drivers of extracellular matrix synthesis. Par4 deficiency exacerbates extracellular matrix degradation through dysregulated MMP-9 activation. Par4−/− mice exhibit significantly elevated pro- and active MMP-9 levels in infarcted regions, enhanced MMP-TIMP(tissue inhibitors of MMPs) interaction, and reduced collagen deposition compared to WT controls by day 7 post-MI, indicative of undermined post- MI cardiac repair.

Crucially, PAR4 governs neutrophil-intrinsic apoptosis rather than macrophage/platelet-mediated mechanism. Despite reduced systemic neutrophil mobilization at day 7 post-MI, Par4^-^/^-^ mice display enhanced intra-infarct neutrophil infiltration, indicating impaired local clearance rather than systemic recruitment. This clearance failure stems from PAR4’s neutrophil-intrinsic role in apoptosis regulation, as demonstrated by impaired caspase-3 activation and reduced TUNEL^+^ cell counts in Par4^-^/^-^ bone marrow neutrophils following thrombin or Par4 agonist peptide stimulation *in vitro*. This anti-apoptotic phenotype translates to delayed neutrophil clearance *in vivo*, with WT mice achieving early apoptotic neutrophil clearance by day 2 post-MI, whereas Par4^-^/^-^ counterparts display delayed clearance until day 7. Mechanistically, this defect correlates with delayed lipoxin A4 (LXA4) dynamics—a pro-resolving mediator—and altered cytokine profiles (↑IL-6/CCL5/CXCL10; ↓IL-4), perpetuating inflammatory cascades.

Adoptive transfer experiments localize PAR4’s functional role to neutrophils: Transferring Par4^-^/^-^ neutrophils into neutropenic recipients exacerbated infarct expansion and ventricular dilation, though less severe than global PAR4^-^/^-^ phenotypes, likely due to incomplete neutrophil replacement or competition with residual WT neutrophils. Conversely, WT neutrophils transfer into Par4^-^/^-^ recipients during acute MI reduced infarct neutrophil infiltration, attenuated hemosiderin deposition, and restored collagen accumulation by day 7, culminating in lower cardiac rupture rates and improved survival. While macrophage efferocytosis drives neutrophil clearance downstream, the available transfer experiments most directly support a neutrophil-intrinsic role for PAR4; impaired inflammation resolution in Par4−/− mice post-MI was not mediated by Par4 expression in macrophages (36). Platelet-specific mechanisms appear less central in these experiments. Adoptive transfer of either wild-type (WT) or PAR4^-^/^-^ platelets comparably attenuated hemosiderin deposition in thrombocytopenic recipients. Both WT and Par4^-^/^-^ platelet transfusions abolished the cardioprotective effects of platelet depletion, mirroring IgG-treated controls in collagen deposition and ventricular dilation, and functional impairment. These findings establish neutrophil-expressed Par4 as a non-redundant regulator of apoptosis-driven inflammation resolution, whose dysfunction underlies maladaptive remodeling in chronic MI.

### 3.3 Nampt inhibition and post-MI cardioprotection: potential links to neutrophil apoptosis

Nicotinamide phosphoribosyltransferase (Nampt), also known as pre-B cell colony-enhancing factor (PBEF) or visfatin, is a 52-kDa enzyme essential for nicotinamide adenine dinucleotide (NAD^+^) biosynthesis. It exists in two forms, with the two overlapping in function. Intracellular Nampt (iNAMPT): Catalyzes the conversion of nicotinamide to nicotinamide mononucleotide (NMN) via the NAD^+^ salvage pathway, maintaining cellular energy metabolism and epigenetic regulation (37). Extracellular Nampt (eNAMPT): Functions as a damage-associated molecular pattern (DAMP) that activates TLR4 signaling, driving pro-inflammatory cytokine release (e.g., TNF-α, IL-1β) (38, 39).

Clinical investigations report acute-phase elevation of serum Nampt levels in AMI patients, with increased concentrations correlating with higher incidence of major adverse cardiovascular events (MACEs) (40). Preclinical isoproterenol-induced MI models corroborate this acute response, showing myocardial Nampt peaking 6–24 hours post-injury—a temporal pattern paralleling troponin T release kinetics (41). Therapeutic neutralization of extracellular Nampt (eNAMPT) using monoclonal antibodies attenuates chronic cardiac inflammation and fibrosis in rodents, as evidenced by reduced collagen deposition and improved left ventricular systolic function at 4 weeks post-MI (38). However, contrasting studies observe myocardial Nampt downregulation under ischemic, ischemia/reperfusion, and pressure overload conditions (42). These discrepancies likely reflect methodological variations, including: 1) differential biomarker interpretation (circulating Nampt as a systemic inflammatory marker versus myocardial Nampt as a local metabolic regulator), 2) temporal sampling windows (acute vs. subacute/chronic phases), and 3) distinct pathological contexts (necrosis-dominant vs. ischemia-reperfusion models). Collectively, the observed variability in Nampt expression highlights its dual roles in post-MI pathophysiology: functioning as a DAMP-driven inflammatory mediator and serving as an NAD^+^-dependent metabolic safeguard.

Nampt exerts anti-apoptotic effects in specific cellular contexts, including cardiac myocytes and immune cells (42, 43), by inhibiting key apoptotic signaling events—including caspase-3 activation and cytochrome c release—through mechanisms distinct from necrotic pathways, as evidenced by unaltered hairpin 2 positivity (42). In neutrophils, inflammatory stimuli (e.g., LPS, TNF-α) induce Nampt synthesis and secretion, thereby delaying apoptosis. This Nampt-dependent survival mechanism is critically validated by antisense oligonucleotide-mediated PBEF (Nampt) knockdown, which abolishes the anti-apoptotic effects of cytokines (IL-1, GM-CSF, IL-8, TNF-α) and restores physiological apoptosis kinetics in septic neutrophils—a hallmark of its regulatory role in clinical sepsis-associated inflammation (43).

Pharmacological inhibition of Nampt with FK866 attenuates neutrophil-mediated injury in I/R models, reducing infarct size and myocardial ROS generation through suppression of CXCL2/CXCL8-driven neutrophil recruitment. Notably, this cardioprotection is neutrophil-dependent, as leukocyte-free models show no benefit (44). Building on its established role in sepsis—where Nampt sustains neutrophil survival via apoptosis inhibition —emerging data implicate similar mechanisms in post-MI inflammation. However, whether myocardial Nampt directly suppresses neutrophil apoptosis (as in sepsis) or acts through divergent pathways (e.g., potentiating recruitment) remains unresolved, necessitating lineage-specific mechanistic studies.

### 3.4 Macrophage efferocytosis: a central mechanism governing apoptotic neutrophil clearance

**Figure 3:**
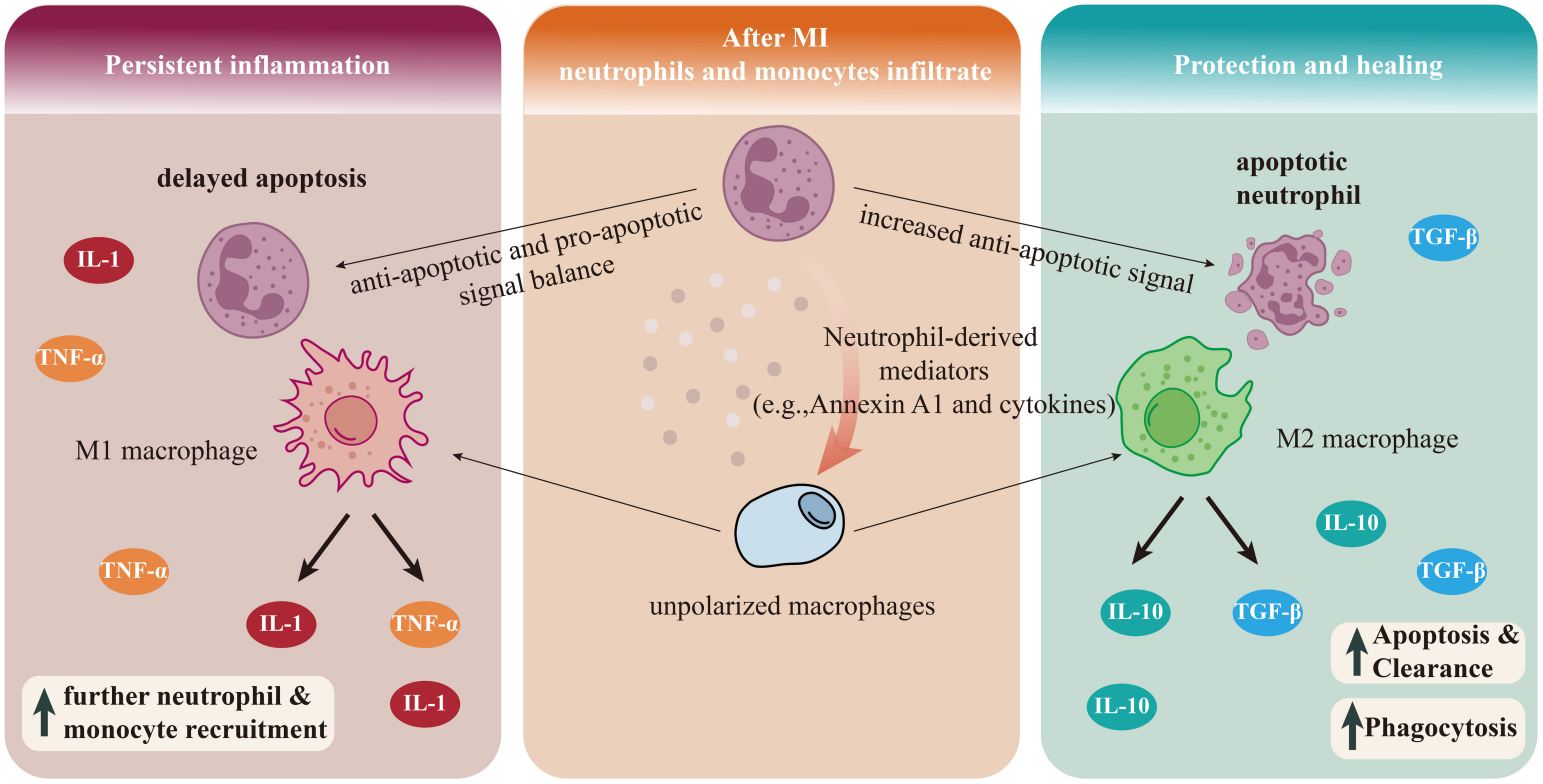
Neutrophil-macrophage crosstalk in post-MI cardiac repair. Following acute MI, neutrophils infiltrate the infarcted myocardium and release neutrophil-derived mediators such as Annexin A1 and cytokines, which modulate macrophage polarization into either pro-inflammatory (M1) or anti-inflammatory (M2) phenotypes. M1 macrophages secrete mediators like IL-1β and TNF-α, whereas M2 macrophages produce tissue-repair factors such as IL-10 and TGF-β. These macrophage-derived signals reciprocally regulate neutrophil apoptosis: timely apoptosis enables efficient efferocytosis by macrophages, triggering the release of anti-inflammatory and reparative mediators (e.g., IL-10, TGF-β) that resolve inflammation and promote tissue repair. Conversely, delayed neutrophil apoptosis synergizes with M1 macrophages to amplify inflammation, perpetuating neutrophil recruitment and macrophage activation. Furthermore, neutrophil-derived mediators shape macrophage phagocytic capacity through phenotypic reprogramming. Dysregulation of this axis—whether due to impaired apoptosis or defective efferocytosis—fuels persistent inflammation, exacerbates cardiac injury, and impedes repair, underscoring the pivotal role of coordinated neutrophil apoptosis and macrophage efferocytosis in post-MI cardiac recovery. MI, myocardial infarction; PS, phosphatidylserine; IL, interleukin; TNF-α, tumor necrosis factor-alpha; TGF-β, transforming growth factor-beta.

The coordinated regulation of neutrophil apoptosis and macrophage efferocytosis constitutes a critical axis for post-MI cardiac repair (**Figure 3**). Post-MI studies support a conserved paradigm in which neutrophils rapidly infiltrate infarct zones to orchestrate microenvironmental priming through secretion of proteolytic enzymes and chemotactic factors, thereby guiding macrophage recruitment (45, 46). Although neutrophils retain intrinsic phagocytic capacity (47), their primary role in cardiac repair lies in creating phagocytic niches for macrophages—which function as specialized effectors tasked with debris clearance and apoptotic cell removal. Apoptotic neutrophils orchestrate their own clearance through two key mechanisms: (1) release of “find-me” chemoattractants such as Annexin A1, and (2) surface exposure of “eat me” signals like phosphatidylserine (PS) (48, 49). PS externalization via activated phospholipid scramblases enables greater macrophage recognition, while plasminogen activator inhibitor 1 (PAI-1) serves as a counterbalancing “don’t-eat-me” signal—evidenced by the increased macrophage phagocytosis of PAI-1 knockout neutrophils compared to WT counterparts (4, 50).

Neutrophil-derived mediators may shape macrophage phagocytic function by modulating their phenotypic reprogramming, with both effects synergistically driving inflammation resolution, wound healing, and functional recovery post-MI. Cardiac macrophages transition from early inflammatory programs to reparative states characterized by efferocytosis, angiogenesis, and scar stabilization, and neutrophil-derived cues participate in this shift during post-MI repair (45, 46). Rather than assigning neutrophils to fixed phenotypic categories, recent neutrophil-diversity studies indicate that tissue cues generate dynamic disease-associated neutrophil states during MI and other inflammatory settings (51–54). These neutrophil-dependent signals can support macrophage reparative maturation through ERK1/2 pathway activation, induction of bridging molecules such as MFG-E8 and Gas6, and transcriptional regulation by PPARγ and Nur77, which collectively enhance efferocytosis and macrophage functional maturation. Conversely, persistent neutrophil-derived inflammatory cues may sustain TNF-α and IL-1β overexpression, creating a microenvironment conducive to chronic inflammation. Critically, neutrophil depletion models demonstrate dysregulated monocyte recruitment and unresolved inflammation (45, 46), thereby underscoring the therapeutic need for balanced immunomodulation strategies that temper acute neutrophil-mediated injury while preserving later macrophage-dependent reparative signaling. Several molecules and pharmacological agents have been shown to modulate the neutrophil-macrophage crosstalk during post-MI repair:

#### 3.4.1 Engineered neutrophil apoptotic bodies reprogram macrophages to promote cardiac repair

The timely efferocytosis of apoptotic neutrophils by macrophages is pivotal for resolving post- MI inflammation and enhancing cardiac repair, as mentioned previously. Capitalizing on this mechanism, engineered neutrophil apoptotic bodies (eNABs) were developed by integrating natural neutrophil apoptotic body membranes (NABMs) to mimic apoptotic neutrophil signaling (55). Both NABMs and eNABs upregulated anti-inflammatory cytokines (TGF-β, IL-10) while suppressing pro-inflammatory cytokines (TNF-α, IL-6), concomitant with increased expression of M2 markers (CD206, Arg1) and modulation of iNOS activity—a phenotype consistent with macrophage reprogramming toward reparative states. To augment therapeutic efficacy, hexyl 5-aminolevulinate hydrochloride (HAL) was encapsulated into eNABs (termed eNABHAL). *In vitro*, eNABHAL outperformed unmodified eNABs (eNABnull), reducing pro-inflammatory factors secretion and boosting the production of anti-inflammatory factors. *In vivo*, eNABHAL-treated rats in a MI model exhibited a favorable immune landscape in infarct border zones: reduced iNOS^+^ pro-inflammatory macrophages, increased CD206^+^ reparative macrophages, and attenuated neutrophil infiltration, without affecting T-cell populations. The enhanced macrophage uptake of eNABHAL drives phenotypic switching, effectively transitioning the infarct microenvironment from destructive inflammation to structured repair. This nanotherapeutic strategy offers a targeted approach to mitigate maladaptive remodeling post-MI by harnessing endogenous repair mechanisms.

#### 3.4.2 FPR2 agonism drives inflammation resolution via coordinated neutrophil apoptosis and macrophage reprogramming

Traditional anti-inflammatory strategies targeting broad suppression of proinflammatory mediator (e.g., glucocorticoids, COX-2/TNF-α inhibitors) carry risks of catastrophic complications including ventricular rupture and elevated mortality. Emerging therapeutic strategies are exploring approaches to enhance inflammation resolution, with formyl peptide receptor 2 (FPR2) agonism representing a promising investigational avenue. This G protein-coupled receptor exhibits ligand-dependent duality: proinflammatory activation via serum amyloid A (SAA)-mediated leukocyte recruitment versus inflammation resolution through lipid mediators like lipoxin A4 that polarize macrophages toward M2-like pro-resolving phenotypes. The selective FPR2 agonist BMS-986235 demonstrates multimodal cardioprotection across species: 1)Neutrophil apoptosis potentiation: In a model of SAA-activated human neutrophils—where the lifespan of proinflammatory neutrophils is prolonged and tissue damage is augmented—BMS-986235 reversed SAA-driven anti-apoptotic effects, reducing viable neutrophils while increasing apoptotic cells, thereby curtailing tissue-destructive neutrophil longevity (56). 2) Macrophage reprogramming: Murine MI models revealed that BMS-986235 increased the proportion of CD206^+^(“M2”) macrophage by day 3 post-infarction without altering total macrophage counts and accelerated neutrophil clearance, evidenced by reduced total neutrophil numbers. 3)Structural & functional preservation: Mouse and rat MI studies showed that BMS-986235 improved LV function and attenuated left ventricular remodeling. Mechanistically, BMS-986235 enhances β-arrestin recruitment and upregulates mediators such as IL-10 and monocyte chemoattractant protein-1, driving neutrophil apoptosis and macrophage-mediated phagocytic clearance. This dual action establishes a self-limiting inflammatory cascade that transitions infarcted myocardium from destructive inflammation to structured repair, offering a paradigm shift in post-MI therapeutics.

#### 3.4.3 Neutrophil-macrophage crosstalk via MerTK enhances efferocytosis to promote cardiac repair

Neutrophil-depleted mice subjected to MI exhibited exacerbated cardiac dysfunction, augmented fibrosis, and progressive heart failure. Paradoxically, despite reduced cardiac expression of M1 macrophage markers and elevated M2 markers, these mice showed significant downregulation of myeloid-epithelial-reproductive tyrosine kinase (MerTK)—a phagocytic receptor hallmarking reparative M2c macrophages essential for apoptotic cell clearance—which correlated with increased TUNEL-positive apoptotic cell accumulation in infarct zones (46). *In vitro* studies confirmed MerTK is predominantly expressed by M2c-polarized macrophages, with minimal expression in M1 and M2a subsets. Exposure to neutrophil secretomes induced M2a-to-M2c polarization through MerTK upregulation, a process mechanistically driven by neutrophil gelatinase-associated lipocalin (NGAL). Furthermore, incubation with neutrophil supernatant or purified NGAL enhanced M2a macrophage efferocytosis capacity—clearance of apoptotic cells—consistent with upregulated expression of the phagocytic receptor MerTK. *In vivo*, histological analysis confirmed neutrophil depletion impaired cardiomyocyte corpse removal, with more apoptotic cells retained in infarcts versus controls. These findings establish neutrophil-derived NGAL as a critical orchestrator bridging neutrophil-macrophage crosstalk to MerTK-dependent efferocytosis, resolving inflammation and facilitating adaptive cardiac remodeling post-MI.

#### 3.4.4 Carprofen disrupts splenocardiac neutrophil-macrophage crosstalk to impede post-MI inflammation resolution

Nonsteroidal anti-inflammatory drugs (NSAIDs) exacerbate MI incidence and elevate heart failure (HF)-related hospital readmissions. However, the precise cellular and molecular mechanisms underlying these NSAID-induced adverse cardiovascular effects remain incompletely characterized. Carprofen (CAP), a model NSAID, was found to exacerbate MI outcomes by disrupting neutrophil-macrophage coordination within the splenocardiac axis—a leukocyte trafficking network essential for inflammation resolution (57). The spleen, as a major leukocyte reservoir, mobilizes neutrophils and monocytes to infarcted myocardium to facilitate repair. Pre-MI CAP treatment primes a pro-inflammatory milieu in the left ventricle (LV), elevating TNF-α, IL-1β, and CCL2 while expanding pre-activated Ly6G^+^ neutrophils and pro-inflammatory F4/80^+^/Ly6Chi macrophages; at baseline, splenic reparative macrophage subsets were not altered, whereas LV reparative F4/80^+^/Ly6Clo macrophages were reduced compared to CAP-negative controls. Post-MI, CAP sustains non-resolving inflammation through amplified “don’t-eat-me” CD47 signaling on splenocardiac neutrophils. This is evidenced by a higher percentage of CD47^+^ neutrophils in the left ventricle (LV) and spleen compared to CAP-negative controls, which inhibits efferocytosis and increases apoptotic cell accumulation (as indicated by elevated TUNEL^+^ cells). Concomitantly, CAP reduces CD169^+^ macrophages critical for splenic innate response, impairing neutrophil clearance and fostering an inversion of the physiological efferocytosis sequence. Image analysis revealed that under CAP-deficient conditions, macrophages phagocytosed annexin V^+^ debris, whereas in CAP-treated groups, neutrophils aberrantly engulfed macrophages, thereby reversing the normal macrophage-mediated neutrophil efferocytosis order. These findings reveal that NSAIDs, exemplified by carprofen, not only fail to resolve post-MI inflammation but actively subvert endogenous resolution programs. By disrupting splenocardiac neutrophil-macrophage coordination—amplifying “don’t-eat-me” CD47 signaling on neutrophils, depleting reparative macrophages, and inverting physiological efferocytosis hierarchies—carprofen paradoxically sustain inflammatory cascades. This impairment of resolution machinery exacerbates cardiac remodeling, highlighting that conventional NSAID use in post-MI settings may inadvertently sabotage the heart’s intrinsic repair capacity rather than providing therapeutic benefit.

## 4 Autophagy: a regulatory node in neutrophil death programming

Autophagy is a conserved cellular process wherein cytosolic components are sequestered within double-membrane vesicles termed autophagosomes, which subsequently undergo lysosomal fusion for macromolecular degradation (58). In cardiomyocytes, basal autophagy serves as a critical homeostatic mechanism, preserving cellular architecture and genomic stability during aging. Beyond its direct cardioprotective roles—such as Nampt-mediated maintenance of cellular energetics through preserving ATP/NAD+ homeostasis which exerts cytoprotection by suppressing cardiomyocyte apoptosis and enhancing autophagic flux (42) —autophagy has been shown to impact immune cell function. This includes modulating neutrophil activity via two complementary autophagy pathways: (1) xenophagy for intracellular pathogen clearance, and (2) PRR-driven non-canonical autophagy that modulates inflammatory responses via NETosis regulation and cytokine control (1).The latter pathway contributes to inflammation resolution and tissue healing during post-MI repair.

Autophagy has also been shown to orchestrate diverse cell death pathways, with its functional roles categorized into two principal mechanisms: (1) autophagy-dependent cell death (ADCD) requiring intact autophagic machinery, and (2) autophagy-mediated cell death (AMCD) wherein autophagic processes initiate alternative cell death modalities (58). Intriguingly, the molecular executors of these autophagic cell death programs may hold prognostic and therapeutic significance in AMI, as evidenced by recent transcriptomic insights into neutrophil-associated autophagy genes (59). Integrated interrogation of Gene Expression Omnibus and human autophagy databases identified differentially expressed autophagy-related genes (DEARGs), with four hub genes (BCL2, MAPK1, RAF1, PRKAR1A) subsequently validated through independent dataset analysis and qPCR experiments. These genes demonstrate regulatory potential within immune cell populations (particularly neutrophils and CD8+ T cells) and show a significant association with AMI progression. AMI specimens exhibited decreased BCL2 expression contrasting with elevated MAPK1, RAF1, and PRKAR1A levels. Notably, BCL2 demonstrated inverse correlation with neutrophil activity, while other DEARGs showed positive associations. Given BCL2’s established role in apoptosis inhibition across cellular systems, these findings suggest potential autophagy-apoptosis crosstalk in neutrophil pathophysiology during AMI.

Evidence also implicates autophagy in NET generation. Platelet-derived polyphosphate (polyP), previously implicated in immunothrombosis through its participation in coagulation cascade activation, has now been mechanistically linked to NET formation via autophagy regulation. Histopathological analysis of arterial STEMI thrombi localized polyP predominantly within platelets adjacent to NET remnants, establishing its role as a platelet-derived NET inducer (21). Mechanistically, polyP released from activated platelets induces NET formation through mTOR-dependent autophagy regulation, wherein mTOR inhibition drives autophagic flux. This mechanistic relationship was further substantiated by bafilomycin A1 treatment—pharmacological inhibition of autophagosome-lysosome fusion abrogated polyP-triggered NET release in control neutrophils, definitively establishing autophagy’s indispensable role in polyP-mediated NETosis.

Beyond polyP, high-mobility group box 1 (HMGB1) represents another mediator that orchestrates NET formation through autophagy modulation in thromboinflammatory contexts. Activated platelets secrete HMGB1 that induces neutrophil autophagy, demonstrated by attenuated platelet-induced autophagy following HMGB1 blockade with BoxA (60). Crucially, HMGB1-mediated NET generation depends on intact Receptor for Advanced Glycation End Products (RAGE). Experiments further validated that competitive HMGB1 antagonism significantly attenuates NET generation, Hmgb1^−/−^ platelets confirmed abrogation of NET induction capacity, while exogenous HMGB1 alone suffices to drive neutrophil commitment to NETosis. Furthermore, exposure of neutrophils to HMGB1 preserves mitochondrial membrane potential, stimulates autophagosome biogenesis, and extends neutrophil survival. Blockade of the autophagic flux reverts platelet HMGB1-elicited NET generation and inhibit the ability of HMGB1 to induce autophagosomes. While HMGB1’s role in autophagy-dependent NETosis is well established, evidence reveals its broader regulatory capacity in apoptosis (61). Neutrophils co-cultured with activated platelets (HMGB1-secreting) exhibited preserved mitochondrial membrane potential and resistance to constitutive apoptosis—the default death pathway of unstimulated neutrophils (60). These findings collectively demonstrate HMGB1’s dual functionality: orchestrating NET formation through autophagy induction while simultaneously suppressing apoptotic cell death.

The pathophysiological interplay between neutrophil autophagy, NETosis, and apoptosis characterized in MI acquires enhanced mechanistic plausibility through its recapitulation in sepsis-induced intestinal barrier injury. Experimental interventions modulating autophagic flux demonstrate mechanistic causality: pharmacological autophagy suppression (chloroquine) or induction (rapamycin) reciprocally regulates neutrophil death modality switching between NETosis and constitutive apoptosis (62). Hesperetin intervention attenuates NET generation via ROS/autophagy axis inhibition, effectively redirecting neutrophil death toward apoptosis. This autophagy-induced phenotypic shift toward NETosis or apoptosis confers intestinal barrier protection during sepsis.

## 5 Platelets: modulators of thromboinflammatory neutrophil death

**Figure 4:**
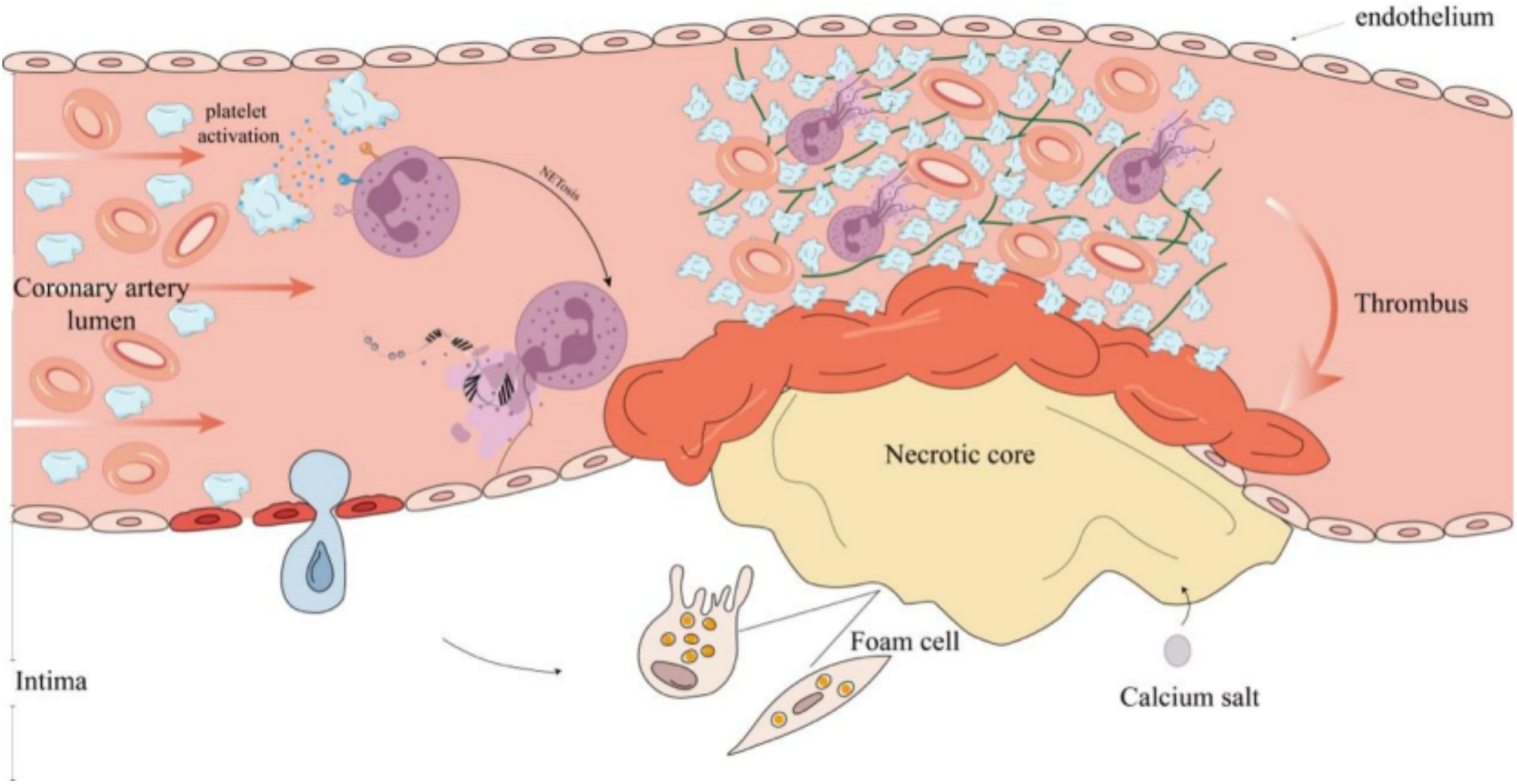
Mechanisms of thromboinflammation: platelet-neutrophil crosstalk and NETosis in coronary thrombus formation. Endothelial injury and reduced blood velocity at sites of coronary artery stenosis promotes neutrophil recruitment and platelet activation. Activated platelets release mediators such as HMGB1, polyP, and CXCL4, which induce neutrophil extracellular trap (NET) formation—a process termed NETosis. NETosis is a distinct form of programmed neutrophil death characterized by chromatin extrusion and release of cytotoxic granular proteins, forming web-like structures termed NETs. These NETs act as scaffolds that bind platelets, fibrin, and coagulation factors, amplifying thromboinflammation. At atherosclerotic plaques, activated platelets, fibrin, red blood cells and NETotic neutrophils accumulate synergistically, driving occlusive thrombus formation. NET-driven thromboinflammation perpetuates a vicious cycle: platelet activation enhances NETosis, while NETs further recruit and activate platelets and neutrophils, exacerbating vascular injury. This interplay underscores the critical role of platelet-neutrophil crosstalk and dysregulated NETosis in coronary thromboinflammatory pathologies. NETs, neutrophil extracellular traps; HMGB1, high mobility group box 1; polyP, Platelet-derived inorganic polyphosphate; CXCL4, C-X-C motif ligand 4.

Activated platelets serve as an important regulator of neutrophil cell death modalities in thromboinflammatory pathologies. In acute coronary syndromes (ACS), platelet activation drives enhanced platelet-neutrophil aggregation via surface receptors including GPIIb/IIIa, GPIbα, and P-selectin, while simultaneously releasing soluble mediators that profoundly alter neutrophil fate. Experimental evidence demonstrates ADP-activated platelets significantly delay neutrophil apoptosis compared to resting platelets, with experiments proving this anti-apoptotic effect stems from soluble platelet-derived factors rather than membrane-bound components (3). Parallel investigations reveal activated platelets supernatants induce NET formation through secreted molecular signals (60). Histopathological correlation emerges from coronary thrombus analyses showing spatial co-distribution of activated platelets, neutrophils, and NET remnants, forming anatomical triads that perpetuate thromboinflammatory cascades (**Figure 4**). This bidirectional crosstalk establishes a self-reinforcing cycle: platelet-derived soluble factors suppress neutrophil apoptosis to sustain inflammatory responses while concurrently programming NET-driven thrombosis, ultimately amplifying ischemic tissue damage.

### 5.1 P-selectin

P-selectin contributes to NETosis through both soluble and membrane-bound mechanisms. Supporting this, circulating neutrophils in P-selectinΔCT/ΔCT mice, which exhibit constitutively elevated soluble P-selectin (sP-sel), display a priming phenotype characterized by increased basal histone citrullination (H3Cit^+^), enhanced surface deposition of P-selectin, and hyperresponsiveness to NETotic stimuli (PAF/ionomycin/PMA). Complementing these observations, recombinant soluble P-selectin (P-sel-Ig) itself induces NETosis. Notably, structural evidence indicates that this activity requires dimerization of soluble P-selectin to achieve the functional avidity necessary for effective engagement of PSGL-1 (P-selectin glycoprotein ligand-1), the primary high-affinity P-selectin counter-receptor expressed on neutrophils. Collectively, these findings establish sP-sel as a thromboinflammatory amplifier that sensitizes neutrophils for accelerated NETosis in pathologies like arterial thrombosis, with dimerization being essential for its pathological signaling (63, 64).

### 5.2 HMGB1

By deploying high-mobility group box 1 (HMGB1), a heparin-binding protein, activated platelets serve as critical regulators of neutrophil fate in thromboinflammatory milieus. This platelet-derived HMGB1 coordinates a tripartite regulatory network: (1) Induction of neutrophil autophagy, as evidenced by the significant attenuation of platelet-induced autophagy when HMGB1 is pharmacologically inhibited using BoxA (a competitive antagonist of HMGB1); (2) Promotion of NETosis, demonstrated by the impaired ability of Hmgb1^-/-^ platelets compared to wild-type controls in generating neutrophil extracellular traps (NETs). This NET-promoting effect is further abolished by pretreatment with HMGB1 receptor-blockers; (3) Suppression of constitutive apoptosis, evidenced by the preserved mitochondrial membrane potential in neutrophils exposed to activated platelets, thereby preventing their default apoptotic pathway (60). Experiments further reveal that HMGB1-mediated NET formation requires autophagy induction, as pharmacological blockade of autophagic flux not only abolishes its ability to stimulate autophagosome formation but also reverses HMGB1-induced NET generation.

### 5.3 polyP

Platelet-derived polyphosphate (polyP) has been identified as a NET inducer in ST-segment elevation myocardial infarction (STEMI). Studies demonstrate that thrombin in infarct-related artery plasma governs the release of polyP from activated platelets, which drives NET formation via mTOR inhibition and autophagy induction. These findings further support the regulatory role of platelet-derived soluble mediators in neutrophil function and thromboinflammatory progression during MI (21).

### 5.4 Oxidized phospholipids

Hematopoietic LNK deficiency (or the human LNK R262W variant reducing function) drives accelerated arterial thrombosis by dysregulating platelet-neutrophil interactions and NETosis. Mechanistically, thrombin-activated Lnk^-^/^-^ platelets exhibit significantly increased surface exposure and secretion of oxidized phospholipids (OxPL) compared to wild-type platelets (65). Concurrently, Lnk^-^/^-^ neutrophils show enhanced priming and responsiveness to OxPL. This dual defect results in markedly elevated NETosis when Lnk^-^/^-^ platelets interact with Lnk^-^/^-^ neutrophils. The central role of platelet-derived OxPL was confirmed by introducing the OxPL-neutralizing agent E06-scFv, which reversed accelerated NETosis, thrombosis in Lnk^-^/^-^ mice. Human relevance was demonstrated using iPSC-derived LNK(TT) cells, which also showed increased NETosis when incubated with LNK(TT) platelet/megakaryocytes compared to isogenic LNK(CC) controls. Clinically, UK Biobank data revealed that individuals carrying both the hyperactive JAK2VF mutation and the LNK R262W allele (which impairs LNK’s inhibition of JAK/STAT signaling) exhibit increased risk of coronary artery disease. Similarly, mice with hematopoietic Lnk^+^/^-^ Jak2VF clonal hematopoiesis showed accelerated thrombosis versus Lnk^+^/^+^ Jak2VF controls. Thus, LNK deficiency may promote thrombosis via platelet-derived OxPL secretion, identifying OxPL blockade as a targeted therapeutic strategy for genetically susceptible individuals.

### 5.5 CXCL4

Chemokines stored in platelet α-granules, such as CXCL4 (platelet factor 4, PF4), exhibit proatherogenic effects by suppressing neutrophil apoptosis and promoting NET formation (66). Experimental evidence demonstrates that platelet deficiency accelerates neutrophil apoptosis in both circulation and splenic compartments. This finding was corroborated by incubating neutrophils with supernatants from mechanically activated platelets (via vigorous agitation), which significantly attenuated apoptosis, whereas supernatants from resting platelets showed no such protective effect compared to spontaneous apoptosis controls. To investigate the role of platelet granule proteins in apoptosis regulation, human neutrophils were cultured with CCL5, PF4, serotonin, or TGFβ. Among these, only PF4 demonstrated dose-dependent anti-apoptotic activity. To establish PF4 specificity, immunodepletion of PF4 from platelet supernatants abolished their anti-apoptotic capacity, confirming PF4 as the principal mediator. *In vivo* validation using a femoral artery ligation model revealed that anti-PF4 antibodies significantly increased Annexin V binding (an apoptosis marker) on circulating neutrophils compared to isotype controls. Notably, this pro-apoptotic effect mirrored observations in platelet-depleted models, underscoring PF4 as the dominant platelet-derived factor governing neutrophil survival (67).

Furthermore, chemokines demonstrate heterophilic interactions, with the CCL5/CXCL4 heterodimer exhibiting enhanced potency in recruiting monocytes and neutrophils. Beyond leukocyte recruitment, this heteromeric complex triggers NET release through direct platelet-neutrophil contact. The engineered inhibitory peptide MKEY, which selectively disrupts CCL5/CXCL4 interactions, effectively suppresses both leukocyte recruitment and NET generation. Experimental validation showed that co-culture of neutrophils with LPS- or TRAP6-activated platelets (but not with soluble LPS/TRAP6 alone) induced robust NET formation, an effect fully abrogated by MKEY treatment. Histopathological analysis revealed near-complete elimination of NETs in MKEY-treated groups, evidenced by drastically reduced citrullinated histone H3 (H3cit)-positive cells within infarct zones compared to controls (68).

These findings collectively establish platelets as important regulators of neutrophil death programming, dynamically coordinating the apoptosis-autophagy-NETosis triad through targeted secretion of mediators like HMGB1, polyP, and CXCL4 to exacerbate ischemic pathogenesis.

## 6 Controversies and future perspectives

Several controversies remain unresolved. First, timing should be treated as a therapeutic variable rather than an afterthought. Early NETosis and protease release may intensify thromboinflammation, endothelial injury, and ECM breakdown, whereas subsequent repair requires neutrophil apoptosis, macrophage efferocytosis, angiogenesis, extracellular matrix organization, and scar maturation. This temporal dependence may help explain why broad anti-inflammatory strategies have produced divergent clinical signals: low-dose colchicine showed benefit in selected post-MI or chronic coronary disease populations in COLCOT and LoDoCo2, whereas the more recent CLEAR SYNERGY/OASIS-9 trial did not significantly reduce cardiovascular events after acute MI (6–8). Similarly, IL-1-directed therapy produced target-specific results: anakinra, a recombinant IL-1 receptor antagonist blocking IL-1α and IL-1β signaling through IL-1R1, mainly reduced inflammatory biomarkers in non-ST-elevation acute coronary syndromes (10), whereas canakinumab, a monoclonal antibody selectively targeting IL-1β, reduced recurrent cardiovascular events at the 150-mg dose in CANTOS but increased fatal infection risk (11). Future interventions may therefore need pathway-selective and time-limited delivery, such as early NETosis attenuation followed by support for apoptotic-cell clearance and reparative macrophage programming (46, 48, 49, 55, 56).

Second, neutrophil heterogeneity may influence therapeutic responses. Post-MI neutrophils likely occupy dynamic states shaped by infarct timing, platelet contact, tissue conditioning, and systemic inflammatory cues rather than stable fixed subsets (51–54). Future studies should therefore define neutrophil death pathways with time-resolved single-cell, spatial, and lineage-specific approaches, ideally linking each neutrophil state to NETosis, apoptosis, autophagy, and downstream macrophage behavior.

Finally, neutrophils act within a systemic immune network that includes bone marrow and splenic myelopoiesis, gut microbiota, platelets, macrophages, endothelial cells, fibroblasts, and injured myocardium (22, 46, 60, 65, 69, 70). MI-induced emergency hematopoiesis and vascular inflammation suggest that local neutrophil death programs may be coupled to systemic leukocyte production and recurrent ischemic risk. Biomarkers that distinguish ongoing thromboinflammation from active resolution may help select patients most likely to benefit from pathway- and time-specific immunomodulation.

## 7 Conclusion

Neutrophil death modalities significantly influence post-MI outcomes by orchestrating the balance between inflammatory injury and tissue repair. NETosis emerges as a thromboinflammatory driver, propagating microthrombosis and infarct expansion through neutrophil extracellular traps (NETs). Conversely, apoptosis constitutes a pivotal mechanism for inflammation resolution, enabling macrophage efferocytosis and reparative signaling to mitigate ventricular dysfunction. Autophagy functions as a regulatory node in neutrophil death programming, fine-tuning the NETosis-apoptosis equilibrium. Crucially, platelets serve as key modulators of neutrophil death modalities, promoting thromboinflammation through the secretion of mediators (e.g., P-selectin, HMGB1, polyP, and CXCL4).

Therapeutic innovations—including PAD4 inhibitors, FPR2 agonists, and eNABs—show promise in selectively suppressing pathological NETosis or supporting the neutrophil apoptosis–efferocytosis axis that drives inflammation resolution. However, challenges persist in balancing acute inflammation suppression with sustained tissue repair, as exemplified by the paradoxical effects of carprofen, an NSAID. Future efforts should prioritize validating death-pathway signatures for prognostic stratification to guide precise interventions, developing therapies that specifically target pathological inflammation without disrupting subsequent repair processes, and engineering integrated multi-target strategies to concurrently regulate opposing death pathways—such as combined NETosis suppression and apoptosis promotion—thereby resolving the injury-repair paradox. Bridging fundamental insights into neutrophil death pathways with clinical translation will be essential for advancing precision immunomodulation to improve long-term cardiac outcomes.
